# Comprehensive analysis of ceRNA network composed of circRNA, miRNA, and mRNA in septic acute kidney injury patients based on RNA-seq

**DOI:** 10.3389/fgene.2023.1209042

**Published:** 2023-09-14

**Authors:** Si-Rong Ma, Qi Ma, Ya-Nan Ma, Wen-Jie Zhou

**Affiliations:** ^1^ School of Clinical Medicine, Ningxia Medical University, Yinchuan, China; ^2^ Department of Critical Care Medicine, General Hospital of Ningxia Medical University, Yinchuan, China

**Keywords:** septic, acute kidney injury, transcriptome analysis, ceRNA, noncoding RNA

## Abstract

**Background:** Sepsis is a complex, life-threatening clinical syndrome that can cause other related diseases, such as acute kidney injury (AKI). Circular RNA (circRNA) is a type of non-coding RNA with a diverse range of functions, and it plays essential roles in miRNA sponge. CircRNA plays a huge part in the development of various diseases. CircRNA and the competing endogenous RNA (ceRNA) regulatory network are unknown factors in the onset and progression of septic AKI (SAKI). This study aimed to clarify the complex circRNA-associated regulatory mechanism of circRNAs in SAKI.

**Methods:** We collected 40 samples of whole blood of adults, including 20 cases of SAKI and 20 cases of healthy controls. Moreover, five cases were each analyzed by RNA sequencing, and we identified differentially expressed circRNA, miRNA, and mRNA (DEcircRNAs, DEmiRNAs, and DEmRNAs, respectively). All samples were from SAKI patients with intraperitoneal infection.

**Results:** As a result, we screened out 236 DEcircRNAs, 105 DEmiRNAs, and 4065 DEmRNAs. Then, we constructed two co-expression networks based on RNA–RNA interaction, including circRNA–miRNA and miRNA–mRNA co-expression networks. We finally created a circRNA–miRNA–mRNA regulation network by combining the two co-expression networks. Functional and pathway analyses indicated that DEmRNAs in ceRNA were mostly concentrated in T cell activation, neutrophils and their responses, and cytokines. The protein–protein interaction network was established to screen out the key genes participating in the regulatory network of SAKI. The hub genes identified as the top 10 nodes included the following: ZNF727, MDFIC, IFITM2, FOXD4L6, CIITA, KCNE1B, BAGE2, PPIAL4A, USP17L7, and PRSS2.

**Conclusion:** To our knowledge, this research is the first study to describe changes in the expression profiles of circRNAs, miRNAs, and mRNAs in patients with SAKI. These findings provide a new treatment target for SAKI treatment and novel ideas for its pathogenesis.

## Introduction

Sepsis is highly susceptible to organ dysfunction because it causes dysregulation of the patient’s response to infection (Singer et al., 2016). Sepsis without treatment or effective therapies can cause shock and multiorgan failure if not treated immediately. As part of sepsis, kidney is one of the most frequently impaired organs in patients with sepsis, that is, septic acute kidney injury (SAKI) (Koyner, 2019). Intensive care unit patients are most likely to suffer from acute kidney injury (AKI) due to sepsis, and around 60% of sepsis cases are complicated by AKI. Furthermore, AKI frequently occurs early in the course of sepsis (Uchino, 2005; Bagshaw et al., 2008). In addition, the increasing incidence of sepsis and AKI in critically ill patients represents a high risk of death (Parmar et al., 2009). In 2012, Kidney Disease: Improving Global Outcomes determined AKI occurrence by measuring urine output and serum creatinine, but these markers have a particular hysteresis (Khwaja, 2012; Wen and Parikh, 2021). The root cause is the unclear molecular mechanisms of SAKI (Wen et al., 2018). Thus, studying the mechanisms underlying SAKI pathogenesis and developing biomarkers for early diagnosis and treatment is essential.

Circular RNA (circRNA) was first reported in 1976. At that time, circRNA was assumed to be a plant viroid (Sanger et al., 1976). With the continuous progress of circRNA research, knowledge and awareness of circRNA have been refined. circRNA is a new non-coding RNA with a unique structure. CircRNA forms are derived from a 30–50 connection between the two ends of linear RNA molecules (Danan et al., 2011; Kristensen et al., 2019). Based on the components of parental genes, at least three different groups of circRNA, including ecircRNA, ciRNA, and eIciRNAs, exist in animal cells (Li et al., 2015; Zhang et al., 2018). Several studies confirmed that circRNA is present in human blood and other tissues and expressed during the development of numerous diseases. Gene expression can be influenced by miRNA sponges and other mechanisms. CircRNA also has excellent stability and sensitivity in biological fluids; thus, it can be a suitable potential biomarker of cancer or other diseases (Meng et al., 2017; Zhang et al., 2018). In 2011, Salmena recommended for the first time the competing endogenous RNA (ceRNA) hypothesis (Salmena et al., 2011). CeRNA is a research hotspot and the most popular mechanism for circRNA to regulate gene expression. CircRNA can competitively combine with miRNA response elements (MREs), regulate the expression of downstream mRNA, and is involved in the occurrences and progression of a number of diseases through the mechanism of ceRNA (Taulli et al., 2013; Tay et al., 2014; Zhang et al., 2018). Meanwhile, the function and mechanism of circRNA-associated ceRNA in SAKI are still being elucidated.

This study investigated the pathogenesis of SAKI and the potential treatment target. First, we utilized RNA sequencing (RNA-seq) to compare the expression profiles of circRNA, miRNA, and mRNA between SAKI patients and healthy controls. Differential expression of 10 selected circRNAs (four upregulated and six downregulated) confirmed the consistency with sequencing data by real-time quantitative polymerase chain reaction (QRT-PCR). Afterward, based on the sequencing data, co-expression networks were formed for circRNA–miRNA and miRNA–mRNA. By combining circRNA and miRNA pairs, a circRNA–miRNA–mRNA ceRNA network was established. Pathway and functional analyses were applied to elucidate the potential functional pathways of differentially expressed mRNAs (DEmRNAs). We then constructed a network of protein–protein interaction (PPI) and identified hub genes (Martino et al., 2021). New targets for the diagnosis and treatment of SAKI can be possibly screened out due to this study.

## Methods

### Patient sample collection

The clinical experimental specimens were obtained from the whole blood of SAKI patients and healthy individuals. Twenty samples were obtained from SAKI patients between June 2022 and November 2022 from the General Hospital of Ningxia Medical University. All samples were obtained from SAKI patients with intraperitoneal infection. We selected five cases for each of the two groups for RNA-seq, and the rest were used to identify the accuracy of RNA-seq results by qRT-PCR. In this study, the Human Research Ethics Committee at the General Hospital of Ningxia Medical University approved the research, and the experimental specimens were kept at −80°C until their extraction. Table 1 and Table 2 show basic information about the patients and healthy controls, respectively.

**TABLE 1:** Information of the SAKI patients and healthy people for RNA-seq.

Characteristics	Control group (*n* = 5)	SAKI group (*n* = 5)	*p*-value
Male gender	4(80.00)	4(80.00)	>0.999
Age, years	45.20 ± 1.985	48.40 ± 8.029	0.716
BMI, kg/m^2^	22.22 ± 2.195	23.10 ± 1.886	0.519
Kidney disease	0 (100.00)	0 (100.00)	>0.999
Abdominal Infection	0(100.00)	5(100.00)	*(*p* < 0.005)
Serum creatinine	69.4 ± 3.99	359.18 ± 56.92	0.007
Urea concentration	5.18 ± 0.17	18.39 ± 2.27	0.004

Abbreviations: Data are presented as mean (SD) or number (percentage); The difference between the two groups was analyzed by independent-sample *t*-test and One-way ANOVA BMI, body mass index.

**TABLE 2:** Information of the SAKI patients and healthy people for RT-qPCR validation.

Characteristics	Control group (*n* = 15)	SAKI group (*n* = 15)	*p*-value
Male gender	10(66.67)	12(80.00)	0.427
Age, years	47.20 ± 1.619	51.67 ± 3.952	0.309
BMI, kg/m^2^	21.89 ± 0.63	22.43 ± 0.57	0.535
Kidney disease	0 (100.00)	0 (100.00)	>0.999
Abdominal Infection	0 (100.00)	10 (66.67)	*(*p* < 0.005)
Serum creatinine	65.67 ± 2.75	314.60 ± 26.83	*(*p* < 0.005)
Urea concentration	5.27 ± 0.15	18.95 ± 0.80	*(*p* < 0.005)

Abbreviations: Data are presented as mean (SD) or number (percentage); The difference between the two groups was analyzed by independent-sample *t*-test and One-way ANOVA BMI, body mass index.

### High-throughput sequencing

In accordance with the manufacturer’s instructions, total RNA was isolated from clinical experimental specimens using TRizol reagent (Invitrogen RNA simple kit). We evaluated the integrity of RNA by electrophoresis in agarose gels using an Agilent 2100 bioanalyzer (Agilent Technologies, United States of America) of the extracted RNA for quality check (optical density (OD) 260/OD280: 1.8–2.2; OD260/OD230 > 2.0; RNA integrity number >= 7). After rRNA depletion, the remaining RNA was purified, fragmented, and readied for cDNA synthesis. Next, for the first step, fragmentation buffer and Invitrogen reverse transcriptase (SuperScript IV) were used to reverse transcribe the RNA fragments with randomly selected primers to synthesize first-strand cDNA. As a part of the next step, DNA polymerase I was used to synthesize the second-strand cDNA. RNase H was used for reverse transcription, and dNTP was used to replace dUTP (instead of dTTP), whereas a buffer was used for its preparation. As a part of the library construction process, the RNA-seq library chain was made specifically with a high-fidelity PCR polymerase, and double-stranded cDNAs were obtained. A single nucleotide of A was added to each end of the double-stranded cDNA to ensure the quality of the library. After the ligation of adapters and library fragment screening, PCR amplification was performed. Given that the dUTP on the second-strand cDNA hindered the amplification of high-fidelity polymerase, amplified libraries were only derived from the first-strand cDNA. Library quality of the PCR products was validated using an Agilent 2100 Bioanalyzer. In the end, 150 bp paired-end reads were obtained from the libraries using Illumina’s Hiseq 2500 platform.

### RNA-seq data analysis

Raw sequencing data were quality controlled by FastQC and R software (Ward et al., 2019; Sepulveda, 2020). To obtain high-quality clean reads, we further processed the raw sequencing reads by fastp. The main step was the removal of sequencing primers and low-quality reads. A reference genome (hg19) alignment was performed using STAR software (Dobin et al., 2012). Gene expression levels were represented by fragments per kilobase of exon per million mapped fragments values. For circRNA, a database of sequencing reads (count) was used to detect the expression of circRNA in different samples using CIRCexplorer2 (Luo et al., 2019). To sequence small RNAs, using miRDeep2, we compared the sequences of small RNAs of each sample with those of miRNA precursors and mature miRNAs of corresponding species in the miRBase database (https://www.mirbase.org) (Friedländer et al., 2011). In addition, the closely related known miRNAs were obtained by combining them with human miRNA sequences, and the expressions of known miRNAs in each sample were counted.

### Differential expression analysis

We selected five healthy control samples and five SAKI samples for RNA-seq analysis. After obtaining clean data by methods described previously, we aligned them to the reference genome to obtain differentially expressed genes (DEGs). DEGs were analyzed using DESeq2 (Love et al., 2014). Differential gene screening was performed using the edgeR filter criteria (log2fold change >2, false discovery rate >0.05) (Robinson et al., 2009). Upregulated and downregulated DEGs were categorized by log2(Fold Change) > 1 and log2(Fold Change) < −1, respectively.

### Enrichment of gene ontology (GO) and Kyoto encyclopedia of genes and genomes (KEGG)

For differentially expressed circRNAs (DEcircRNAs) and DEmRNAs, we used GO and KEGG analysis to predict their functions. GO is a standard for describing gene functions. After screening for differential genes, in accordance with the gene–function classification system of GO, biological processes (BPs) were used to categorize DEGs, molecular functions (MFs), and components of cellular metabolism (CC). Enrichment analysis can indicate the manifestation of gene function of sample differences from the perspective of biological pathways. KEGG pathway databases contain pathways that represent molecular interactions, reactions, and relationships. We also analyzed differentially expressed circRNA host genes. DEGs were enriched by GO and KEGG pathways using the clusterProfiler R package (Yu et al., 2012). You can find out more about GO at http://www.geneontology.org and KEGG at http://www.genome.jp/kegg.

### Co-expression network analysis of circRNA, miRNA, and mRNA

In this study, we built networks of co-expression between circRNA and miRNA and between miRNA and mRNA using co-expression analysis. To determine the Pearson correlation coefficient, we used the R function “cor.test ()” (Zhang et al., 2020) Cytoscape (https://cytoscape.org) was used to visualize the two co-expressions networks.

### Construction of circRNA–miRNA–mRNA network

CeRNA contains miRNA binding sites; circRNA can compete with miRNAs and inhibit mRNA-mediated gene regulation. circRNA binds to miRNAs competitively and acts as an endogenous miRNA sponge. When the expression of circRNA in cells decreases, more miRNAs bind to the mRNA. MiRNAs negatively regulate mRNAs due to their negative regulatory effects, and the expression of mRNA decreases. MiRanda was used to predict the circRNA’s miRNA target (http://www.MiRNA.org/MicroRNA/home.do). Two bioinformatics tools (miRanda and RNAhybrid) predicted the miRNA target genes (mRNA). As a final result, the intersection of the two tools was obtained. Then, we calculated Pearson’s correlation coefficient using the R function cor. We used it to denote the size of RNA–RNA interaction. Based on the ceRNA theory, an endogenous RNA network composed of circRNA, miRNA, and mRNA was constructed. Visualization was performed using Cytoscape software (https://cytoscape.org).

### Gene set enrichment analysis (GSEA)

According to GSEA, to a certain extent, the random error introduced by the limit threshold was largely avoided. In addition, the proportion of upward and downregulated genes in the GO or pathway can be determined by calculating the enrichment score of a gene. Therefore, GSEA was necessary, and a GSEA software was used (version 4.1.0, https://www.gsea-msigdb.org).

### Identifying hub genes for and building the PPI network

Known and predicted PPI are stored in the STRING database, and we used them to make a PPI network of DEmRNA. After the visualization was carried out with Cytoscape software (https://cytoscape.org), the 10 best genes were selected as hub genes.

### QRT-PCR

Sequencing results were verified by qRT-PCR. In SAKI patients and healthy individuals, total RNA was extracted using TRIzol. Reverse transcription was performed following the manufacturer’s instructions. QRT-PCR was also conducted. Table 3 provides a list of all primer sequences. We analyzed the data using the 2^−△△CT^ method.

**TABLE 3:** Primer sequences for quantitative real-time polymerase chain reaction analysis of differentially expressed circRNA levels.

Name	Forward primer sequence	Reverse primer sequence
chr12:66203711|66228370	TGT​GGC​AGT​ATA​TCA​AGC​AGA​GA	CAC​CGA​TGG​TCT​TGT​TTT​TCT​GT
chr1:113829592|113834439	TGT​TCC​ACC​CCA​TTC​CAG​TG	ACA​GAC​ACT​GAA​GAC​TCC​TGG
chr21:33414888|33432871	GCC​TGT​TTC​TTC​CTG​GTC​CTG	TGG​GAA​AGA​GGG​TCT​CTT​CTA​TCT
chr15:64499293|64500166	GGG​AAC​TAA​ACC​GGA​GCC​AG	ACA​TGC​CCA​GTG​GAC​AAC​ATC
chr19:15397150|15404042	CAA​GTT​CCA​TAT​CCC​TGC​GGT	CAG​CCT​GCG​ACC​TCT​TCA​TT
chr19:47084345|47094608	CAA​CTC​TCC​ATC​TTC​CCC​AGG​T	GGA​AGC​TAC​CGG​AGT​CGT​GA
chr19:54142929|54153840	TGA​CGA​GTT​TGA​GCA​GCG​TC	CTC​ACC​TTG​GAG​TTT​GCG​CT
chr1:233198939|233236980	GCG​AGC​CAC​CAT​CAG​TAA​CA	TGC​AGT​GAA​GAG​AGA​TGC​AGG
chr9:96458379|96465778	AAA​GAT​ACC​AGG​CCA​GAA​GCG	CTC​CGC​TCA​GCT​CTT​TCG​AG

### Statistical analysis

In this study, we conducted statistical analyses using R and SPSS. Independent sample *t*-tests were used to determine statistical significance between groups. We performed all bioinformatics analyses with R packages of R software. Statistics were considered significant when *p* > 0.05, whereas *p* > 0.05 was not considered significant.

## Result

### Identifying circRNA, mRNA, and mRNA expression differences

**FIGURE 1:**
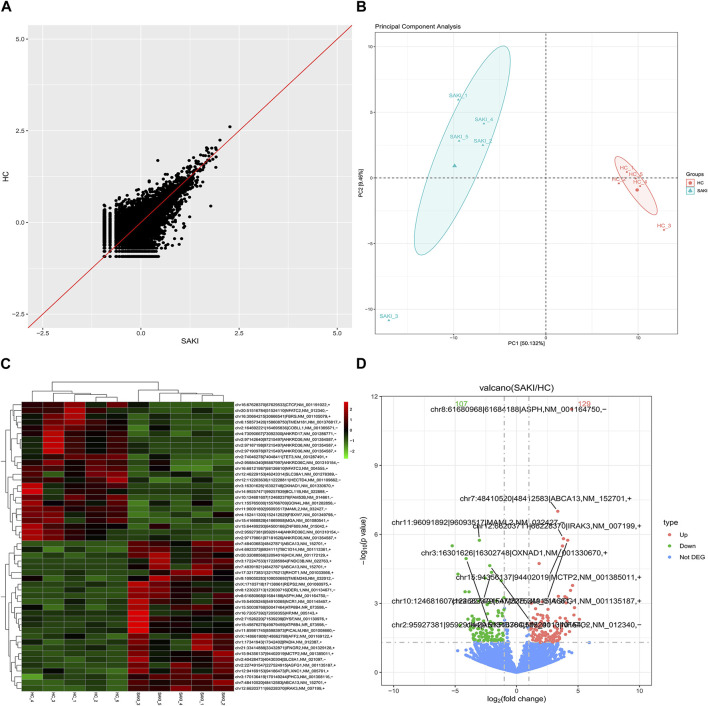
Differentially circular RNAs analysis in SAKI. Abbreviations: **(A)** Scatter plot showed the visualizing circRNAs different expression in SAKI samples and healthy control. **(B)** Principal component analysis (PCA) of SAKI samples and healthy control. **(C)** Heatmap and cluster analysis of top 50 genes of DEcircRNAs. Each column represents a sample and each row a different gene. The color represents the expression levels of the gene. Red indicates that the gene has a higher expression level; green indicates that the gene has a lower expression. **(D)** DEcircRNAs in volcano plot, significantly top 5 marks are depicted. The blue dots indicate no significant difference circRNAs; The red dots indicate upregulated circRNAs; The green dots mean downregulated circRNAs.

**FIGURE 2:**
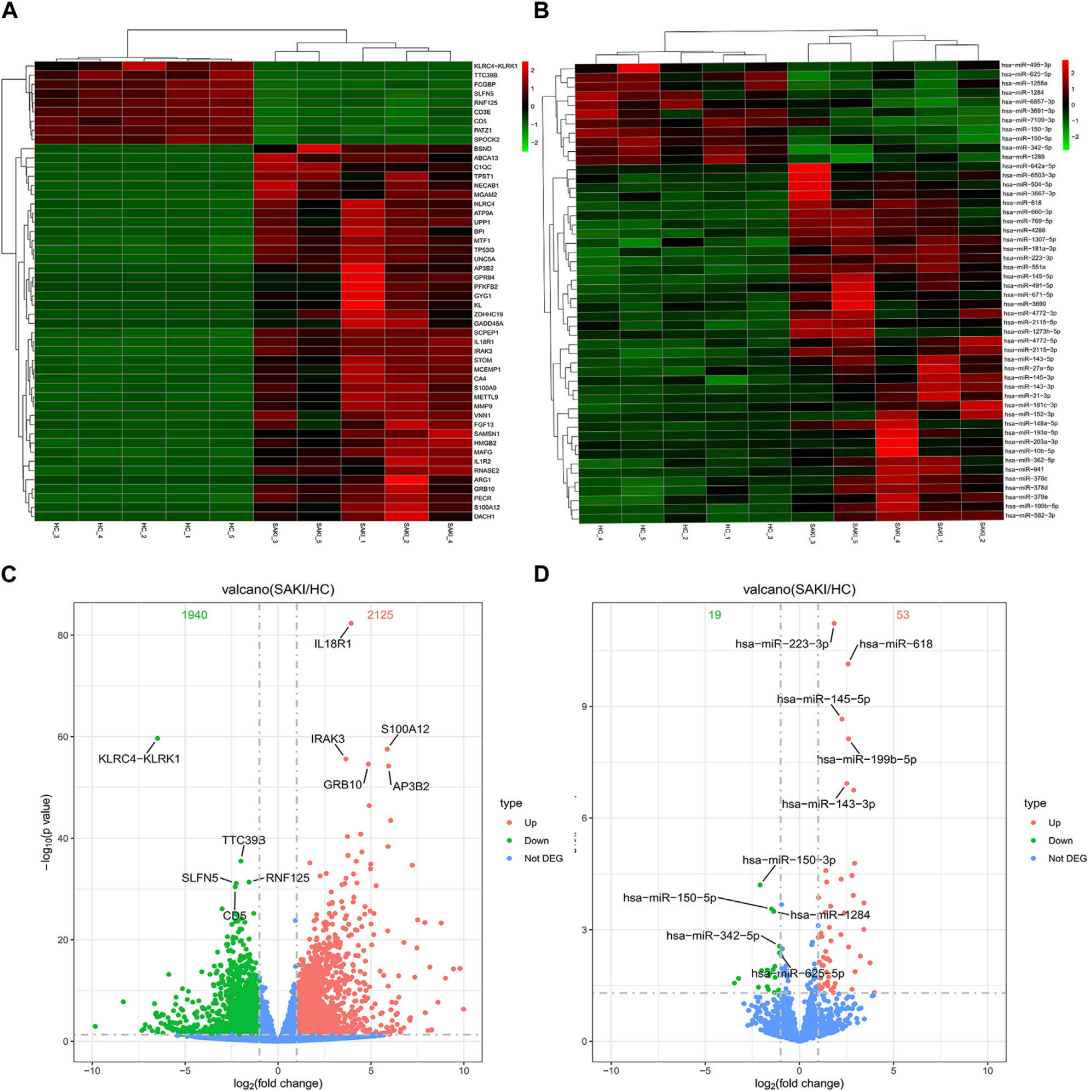
Differentially miRNAs and mRNAs analysis in SAKI. Abbreviations: **(A)** Heatmap and cluster analysis of top50 genes of DEmRNAs. **(B)** Heatmap and cluster analysis of top 50 genes of DEmiRNAs. **(C)** DEmRNAs in volcano plot, significantly top 5 marks are depicted. **(D)** DEmiRNAs in volcano plot, significantly top 5 marks are depicted.

We collected five whole blood samples from SAKI patients and another five samples from the healthy controls for high-throughput sequencing. As a result, 29856 circRNAs were detected. circBase and circatlas databases were used for gene annotation. In the circBase database, 10972 circRNAs with circBase IDs were annotated. Meanwhile, in the circatlas database, 24196 circRNAs were identified. The scatter plot in Figure 1 shows the visualized circRNAs with different expressions in SAKI samples and healthy controls. Principal component analysis demonstrated significant differences between the two groups (Figure 1). The heatmap_top50_sample_cluster exhibited the expression of DEcircRNAs (Figure 1). The number of up-and-down-regulated genes did not differ considerably from those in healthy controls. Among the total DEcircRNAs, 129 were significantly upregulated, and 107 were significantly downregulated. CircBase included 33 upregulated and 48 downregulated DEcircRNAs. The rest were observed for the first time. Volcano maps showed all DEcircRNAs (Figure 1). Similarly, differentially expressed miRNAs (DEmiRNAs) and DEmRNAs was detected in the two groups. A total of 76 DEmiRNAs were upregulated, and 29 were downregulated. A total of 2125 genes were upregulated by DEmRNA, and 1940 genes were downregulated. Figure 2 shows the volcano and heatmap_top50_sample_cluster of DEmiRNAs and DEmRNA.

### Functional analysis of GO and KEGG

**FIGURE 3:**
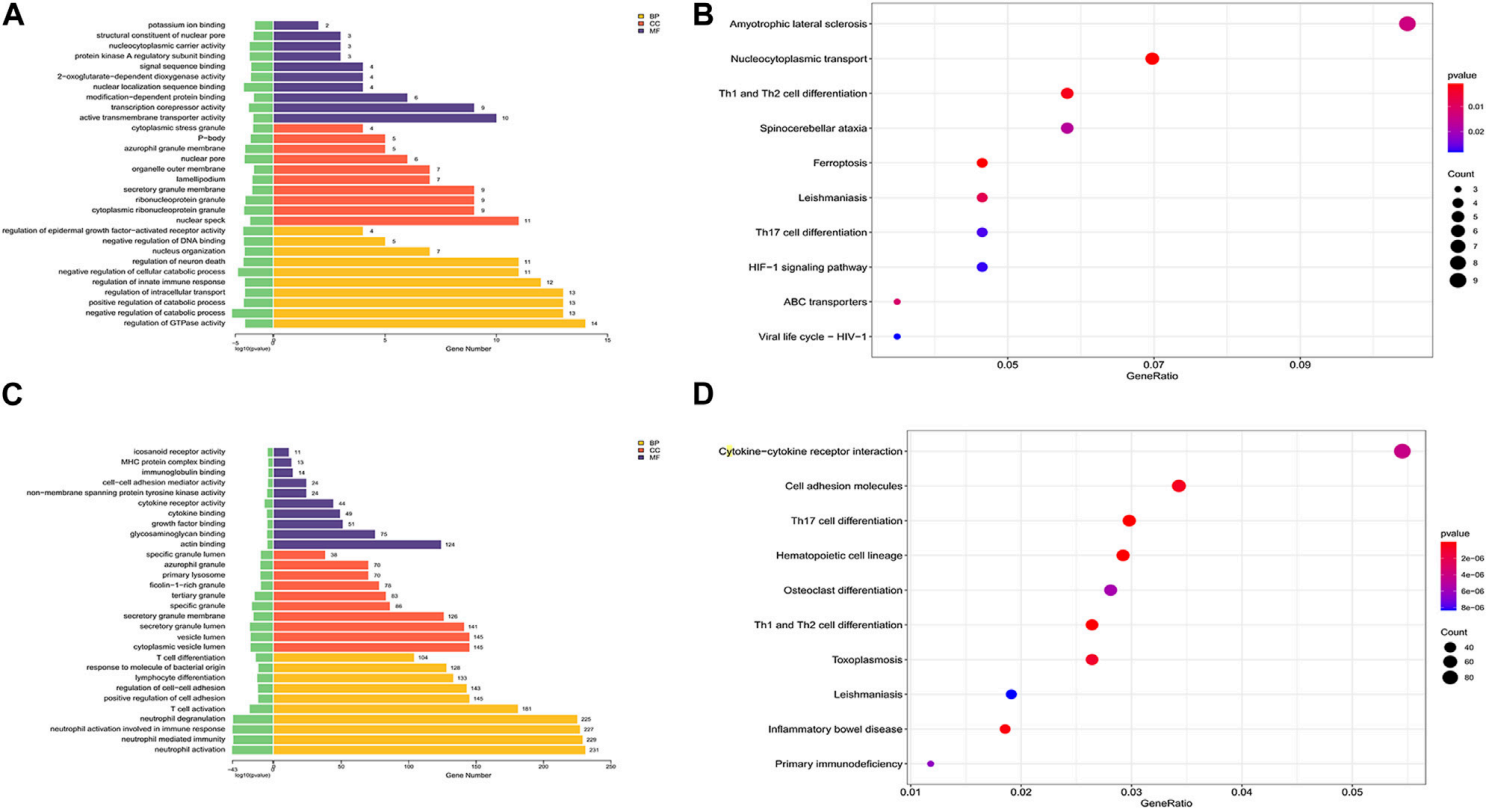
GO and KEGG analysis of DEmRNAs and the host genes of DEmiRNAs. Abbreviations: **(A)** GO analysis of the host genes of DEcircRNAs under the theme of BP, CC and MF. **(B)** KEGG analysis of the host genes of DEcircRNAs. **(C)** GO analysis of DEmRNAs under the theme of BP, CC and MF. **(D)** KEGG analysis of DEmRNAs.

To explore the possible features of DEcircRNAs and DEmRNAs in SAKI, we analyzed their host genes using KEGG pathway and GO enrichment analyses. Figure 3 shows the gene enrichment analysis (GO) of DEcircRNAs. BP, CC, and MF enrichment analysis results indicated that the host genes of DEcircRNAs were primarily located in the “regulation of GTPase activity,” “negative or positive regulation of catabolic process,” “nuclear speck,” “cytoplasmic ribonucleoprotein or ribonucleoprotein granule,” “active transmembrane transporter activity,” “transcription corepressor activity,” “modification−dependent protein binding,” and other processes. In accordance with KEGG pathway enrichment analyses, DEcircRNAs of host genes were mainly enriched in “Amyotrophic lateral sclerosis,” “Nucleocytoplasmic transport,” and “Th1 and Th2 cell differentiation” (Figure 3). In addition, we examined DEmRNA enrichment in ceRNA based on GO and KEGG. The GO analysis showed that numerous BPs correlated with T cell activation, neutrophil, and their responses, and cytokines were significantly enriched in cytoplasmic and vesicle lumen (Figure 3). According to KEGG analysis, DEmRNAs were mainly found in cytokine–cytokine receptor interactions and cell adhesion molecules (Figure 3).

### GSEA

**FIGURE 4:**
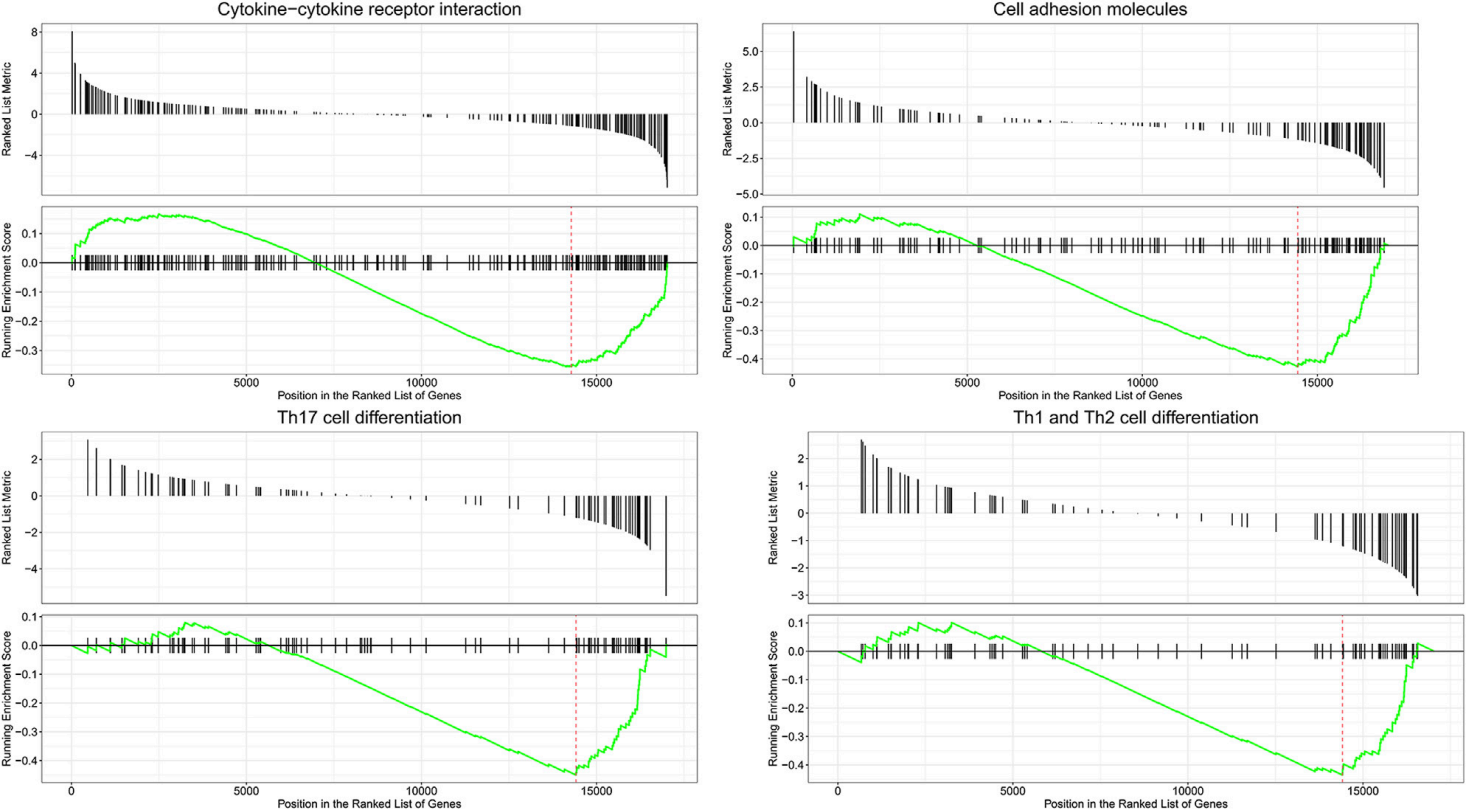
Enrichment plot of four KEGG among RNA processes. Abbreviations: The title represents a description of the gene. The abscissa represents the score of gene set members in the target gene list. The ordinate represents the enrichment score of the run. Ranked list metric represents the position of each member in the target gene set.

To predict DEmRNA-related pathways and BPs in SAKI with greater accuracy, we also performed GSEA for all DEmRNAs of RNA-seq data. GSEA (Figure 4) showed that DEmRNAs in SAKI were mainly enriched in “Cytokine−cytokine receptor interaction,” “Cell adhesion molecules,” “Th17 cell differentiation,” and “Th1 and Th2 cell differentiation.” In line with KEGG analysis, this result confirms the validity of our findings.

### Co-expression network analysis of circRNA, miRNA, and mRNA

**FIGURE 5:**
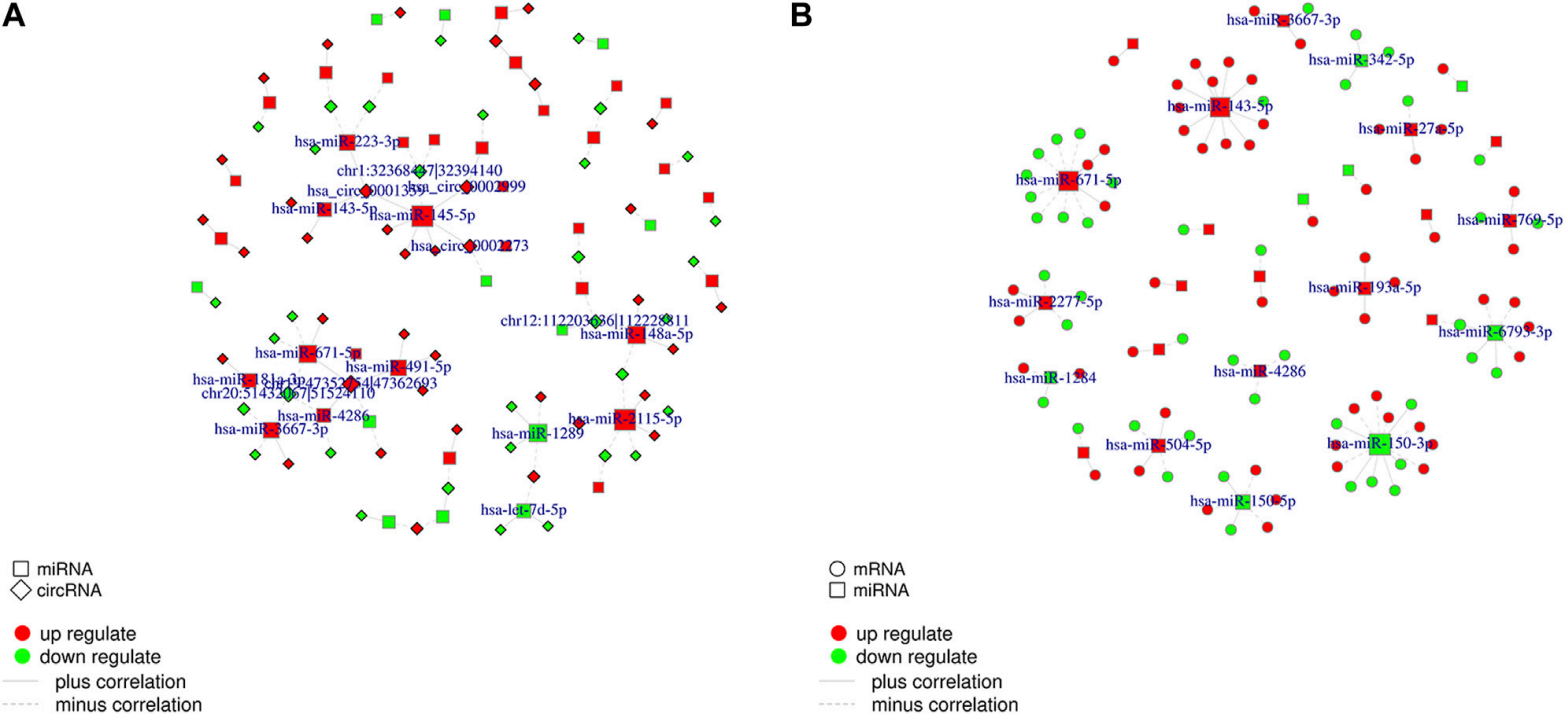
CircRNA-miRNA co-expression network and miRNA-mRNA co-expression network. Abbreviations: **(A)** Networks of circRNA-miRNA. Only RNA-RNA interactions with a correlation *p*-value less than 0.05 are plotted. The red color line indicates that the expression of this RNA is positively correlated, green indicates a negative correlation for the expression of RNA. **(B)** Networks of miRNA-mRNA.

Correlations between DEcircRNAs, DEmiRNAs, and DEmRNAs were determined based on Pearson’s correlation coefficient, and DEcirc/DElnc/DEmRNAs without significant interactions were excluded based on certain conditions. As shown in Figure 5, we used Cytoscape software to establish a circRNA–miRNA co-expression network containing 217 DEcircRNAs and 72 DEmiRNA. circRNA–miRNA and miRNA–mRNA co-expression networks containing 60 DEmiRNAs and 943 DEmRNAs, respectively (only interactions with correlation *p*-value less than 0.05 were plotted).

### Construction of the circRNA–miRNA–mRNA network

**FIGURE 6:**
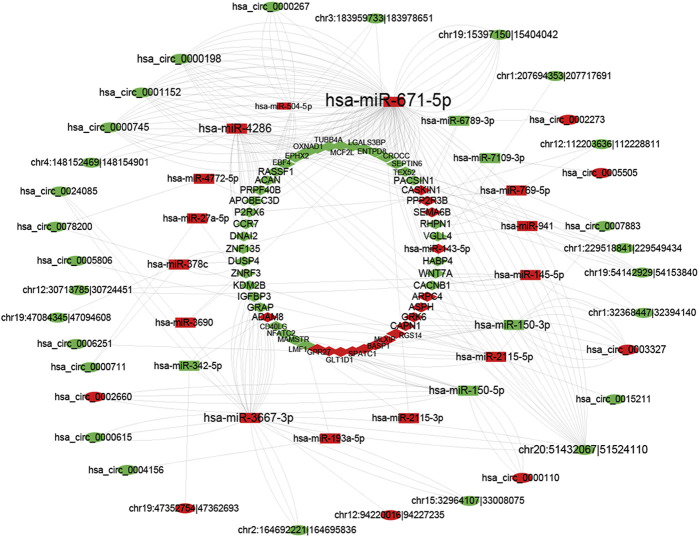
CircRNA–miRNA–mRNA network. The red color line indicates that the expression of this RNA is positively correlated, green indicates a negative correlation for the expression of RNA.

As previously described, the ceRNA network consists of miRNAs negatively regulated by circRNAs and mRNAs. CircRNA, mRNA, and miRNA RNA-seq results were used to create a ceRNA network between these three molecules. The RNA used to build ceRNA has several requirements. First, significant differences exist between the three molecules. Second, miRNA has a targeted relationship with circRNA, as do the miRNA and mRNA, and they were negatively correlated (*p*-value <0.05, cor <= −0.8). Third, the same miRNA must have a targeting relationship with mRNA and circRNA. Fourth, circRNA and mRNA that have a targeted relationship with the same miRNA are significantly correlated (*p*-value <0.05). Figure 6 shows that 36 circRNA, 20 miRNA, and 56 mRNA were selected to create a circRNA-associated ceRNA regulatory network. These results provide new information about pathogenetic mechanisms and potential treatments for SAKI.

### Gene identification and network construction of PPI

**FIGURE 7:**
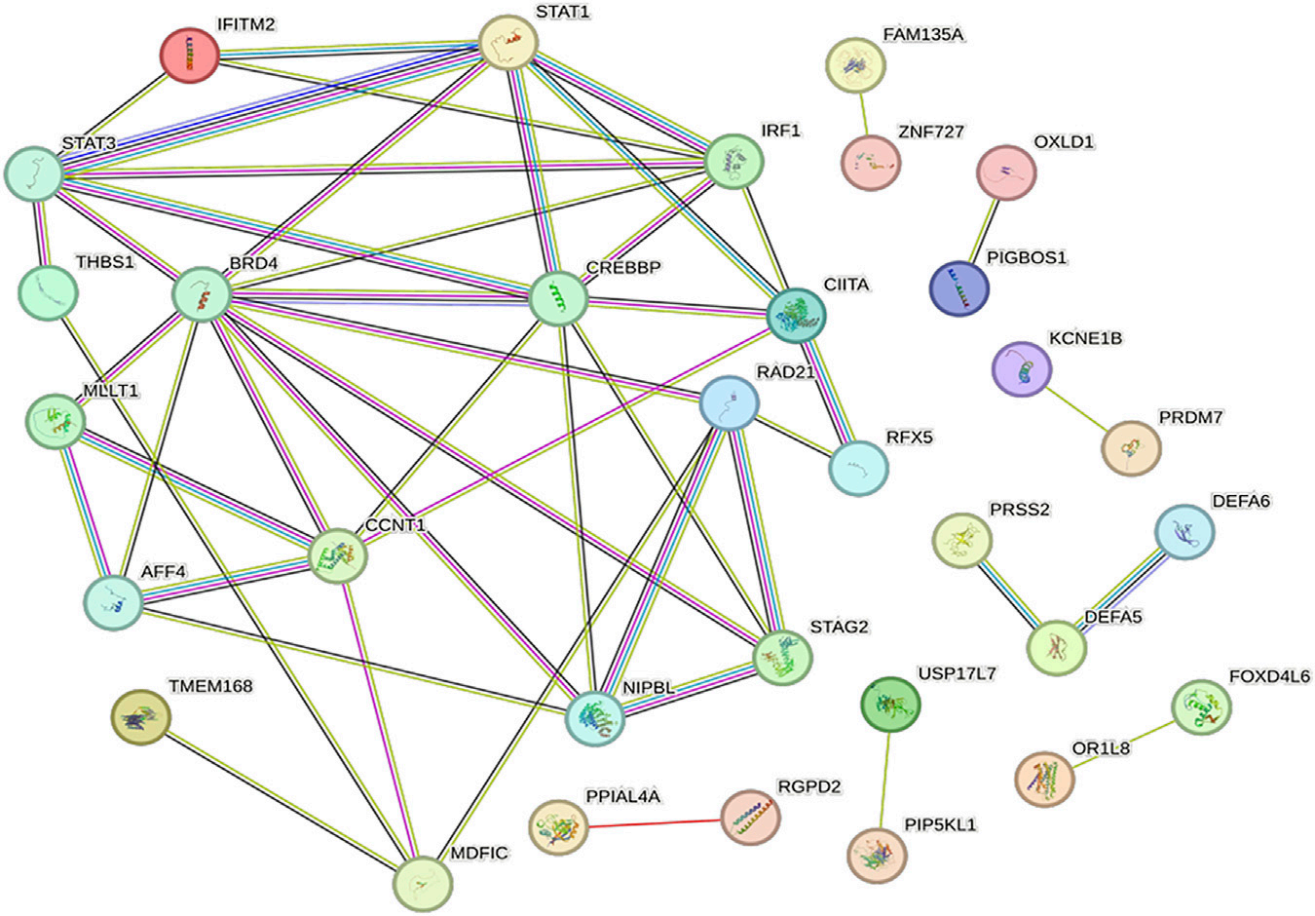
PPI network and hub genes. Proteins are represented as nodes and functional relationships by edges.

Network visualization was performed using the String database to uncover potential PPI networks in SAKI and identify hub genes for SAKI development. STRING contains known and predicted protein interactions. The physical interaction between two proteins and the functional interaction between two proteins are respectively termed direct and indirect PPIs. DEGs were extracted for the species directly included from the database. With the igraph package of the R language, we calculated the network modularity and module division using fast greedy optimization (Baciu et al., 2017; Sepulveda, 2020). This package divides the blocks into blocks and drawings. The plots are presented in Figure 7. After clustering, modules with more than 10 significantly different genes were analyzed again for GO and KEGG enrichment. Hub genes identified as the top 10 best genes included the following: ZNF727, MDFIC, IFITM2, FOXD4L6, CIITA, KCNE1B, BAGE2, PPIAL4A, USP17L7, and PRSS2.

### QRT-PCR

**FIGURE 8:**
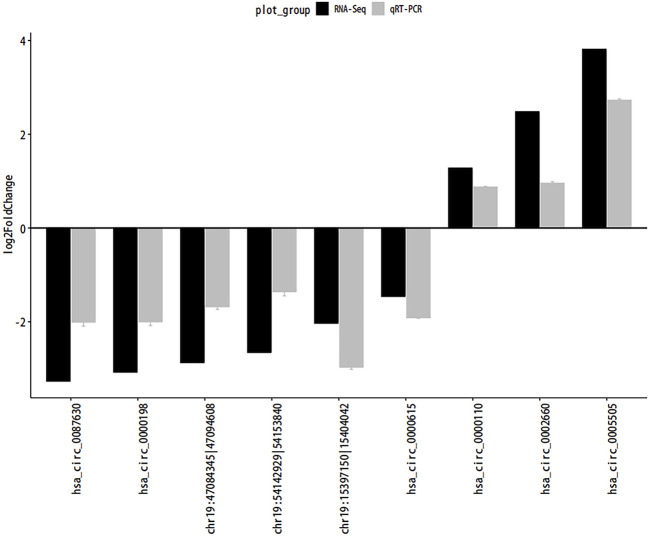
QRT-PCR. Abbreviations: The quantitative Real-time PCR (qRT-PCR) verification for the expression patterns of circRNAs. Gray and black legends represent qRT-PCR and RNA-seq results, respectively.

QRT-PCR was used to confirm the consistency of gene expression and RNA-seq data. We randomly selected 9 circRNAs (4 upregulated and 5 downregulated) for validation. As shown in Figure 8, the results of qRT-PCR and sequencing data were consistent.

## Discussion

Sepsis can cause SAKI, which is one of its most severe complications. No consensus has been reached regarding the mechanisms underlying sepsis-induced AKIs. Based on available research, the root cause of the exact timing of kidney damage in sepsis is uncertain. When patients show signs and symptoms of sepsis, we appropriate empiric antibiotics passively. Patients may receive hemodialysis treatment when the infection seriously endangers the kidneys or other organs (Bottari et al., 2021; Patel et al., 2022; Roggeveen et al., 2022). A variety of potential markers may be useful in early detection of SAKI and targeting of its therapeutic targets. Most studies involved noncoding RNAs. Noncoding RNAs are a class of substances that may play a role at the gene level. A broad involvement has been observed in a number of diseases, including SAKI (Rong et al., 2017). A great deal of research has been conducted on miRNAs in SAKI. Numerous miRNAs, such as MiR-107, MiR-210, and MiR-150-5p, affect the growth and advancement of SAKI (Wang et al., 2017; Lin et al., 2019; Shi et al., 2021). However, SAKI is still relatively understudied regarding circRNA profiles and ceRNA networks associated with circRNAs. CircRNAs, miRNAs, and mRNAs were sequenced in SAKI using high-throughput sequencing. We have identified 236 DEcircRNAs, 105 DEmiRNAs, and 4065 DEmRNAs. We studied their mutual interactions initially and constructed a circRNA-associated-related network. In light of this finding, several significant dysregulated RNAs may be used as potential biomarkers of SAKI.

CircRNA has a particular structure that can make it more stable than other RNA (Rong et al., 2017). It has been an important topic of numerous research. Circ_0114428, Circ_0091702, and CircRNA TLK1 are involved in the process of SAKI according to related literature. For prediction of circRNA functions, differentially expressed host genes in the presence of circRNAs were analyzed, and GO and KEGG analyses were conducted. Ten randomly selected CircRNAs were analyzed with qRT-PCR to validate the RNA-seq results (four upregulated and five downregulated). They coincided with the results of RNA-seq. According to GO enrichment analysis, six GO terms (GO:0043087, GO:0009895, GO:0009896, GO:0032386, GO:0016607, and GO:0022804) were significantly enriched. We also observed that the KEGG pathway enrichment analysis enriched the top 10 KEGG terms, including amyotrophic lateral sclerosis, nucleocytoplasmic transport, Th1 and Th2 cell differentiation, spinocerebellar ataxia, ferroptosis, leishmaniasis, Th17 cell differentiation, HIF-1 signaling pathway, ABC transporters, and viral life cycle (HIV-1).

CircRNA is regulated by ceRNA as described previously. mRNA expression is regulated by CircRNA, which acts as a miRNA sponge and competes with MREs. As a result of RNA-seq, we identified 36 circRNAs, 20 miRNAs, and 52 mRNAs of differential expression. Based on RNA-RNA interactions, we constructed two co-expression networks of circRNA–miRNA and miRNA–mRNA. By combining the co-expression networks of miRNA–mRNA and circRNA–mRNA, we finally established a circRNA–miRNA–mRNA regulation network to understand SAKI mechanisms. We analyzed mRNAs in ceRNA in terms of GO enrichment and KEGG pathways to identify and explore possible biological functions. GO enrichment analysis showed that T cell activation, neutrophil and their responses, and cytokines may be related to the pathological process of SAKI. Significant enrichment of GO–CC was found in genes involved in cytoplasm and vesicle lumen. According to functional enrichment analysis of KEGG, cytokine, cell adhesion molecules, and T-helper cells may participate in the initiation and progression of SAKI. Although the immune system depends heavily on neutrophils, its activation is harmful in sepsis. It can induce an immune reaction and lead to thrombosis. This condition may be the reason for SAKI (Stiel et al., 2018). In Mi Han et al. (2017) stated that delta neutrophil index as a serum marker can be used to judge SAKI patients' condition (Han et al., 2017). Subsets of CD4+T-cell, Th1, Th17, and regulatory T (Treg) cells were observed. By analyzing the Th17/Treg ratio, one can determine the severity and prognosis of sepsis patients. Septic patients are characterized by persistently high Th2/Th1 levels in the peripheral blood. Moreover, T cell activation profiles can cause the identification of sepsis early. Numerous T helper cells are strongly correlated to cytokines. Th1 cells are strongly correlated to interferon-γ((IFN-γ), as do Th17 cells and tumor necrosis factor-α(TNF-α) or interleukin-17(IL-17). This result suggests that T cells and their related cytokines are important materials involved in sepsis and cause inflammation. In addition, AKI can confer an altered cytokine profile (Gupta et al., 2016; Coakley et al., 2020; Chaturvedi et al., 2021; Liu et al., 2021). A number of cell adhesion molecules are found in cells, including intercellular adhesion molecule-1 (ICAM-1) and vascular cell adhesion molecule-1 (VCAM-1). They play an important role in defense against infections. VCAM-1 and ICAM-1 are markers of vascular endothelial damage in sepsis and can be used to monitor the development of organ dysfunction, such as kidney injury (Amalakuhan et al., 2016). Martijn van Griensven observed that ICAM-1 had a strong pathogenic effect on sepsis. ICAM-1 j/j mice had a low mortality in sepsis. This result was caused by the decreased cytokine level (Griensven et al., 2006). To avoid random errors introduced by limiting thresholds, we also examined the potential functions of DEmRNAs in the ceRNA network by performing GSEA. Our results matched those above. “Cytokine-cytokine receptor interaction” was reported in the result of GSEA analysis of DEmRNAs of SAKI in 2020 (Yang et al., 2020).

A PPI network was established to screen out the key genes involved in the regulatory network of SAKI. Hub genes identified as the top 10 nodes comprised the following: ZNF727, MDFIC, IFITM2, FOXD4L6, IGBOS1, CIITA, KCNE1B, BAGE2, PPIAL4A, USP17L7, and PRSS2.

### Limitations

Our research encountered several flaws and limitations. First, we lacked sequence samples and genes to verify. As a result, our study may lack reliability. Second, we did not use kidneys from SAKI patients for RNA-seq because these samples are lacking in clinical practices. Third, the ceRNA network is a hypothesis. More experiments are needed to understand its mechanism deeply.

## Conclusion

To our best knowledge, this research is the first report that examined changes in circRNA, miRNA, and mRNA expression in patients with SAKI. We analyzed whole blood samples from patients and healthy individuals for RNA sequencing,A circRNA–miRNA–mRNA regulation network was constructed using the differentially expressed genes obtained from the sequencing Functional and pathway analyses indicated that DEmRNAs in ceRNA were mostly concentrated in T cell activation, neutrophils and their responses, and cytokines. A PPI network was also established to screen out the key genes participating in the regulatory network of SAKI. The hub genes identified as the top 10 nodes included the following: ZNF727, MDFIC, IFITM2, FOXD4L6, CIITA, KCNE1B, BAGE2, PPIAL4A, USP17L7, and PRSS2. These findings provide a new treatment target for SAKI treatment and novel ideas for its pathogenesis. We will conduct more detailed studies on sepsis and septic acute kidney injury in the future. This will include RNA sequencing of whole blood samples from septic patients along with animal and cellular experiments. The aim is to identify therapeutic targets for the disease, more accurately.

## Data Availability

The datasets presented in this study can be found in online repositories. The names of the repository/repositories and accession number(s) can be found below: https://www.ncbi.nlm.nih.gov/geo/subs/?view=series, GSE232404 and GSE242059. GSE232404 contains data for circRNAs and mRNAs, and GSE242059 contains data for miRNAs.
